# Identification and Validation of qRT-PCR Reference Genes for Analyzing *Arabidopsis* Responses to High-Temperature Stress

**DOI:** 10.3390/cimb46120857

**Published:** 2024-12-18

**Authors:** Siyu Chen, Qi Cai, Peipei Liu, Jingru Liu, Guanzhou Chen, Hanwei Yan, Han Zheng

**Affiliations:** 1 National Engineering Laboratory of Crop Stress Resistance Breeding, School of Life Sciences, Anhui Agricultural University, Hefei 230026, China; 13721170090@163.com (S.C.); cq18726805780@163.com (Q.C.);; 2 Laboratory of Modern Biotechnology, School of Forestry and Landscape Architecture, Anhui Agricultural University, Hefei 230026, China

**Keywords:** *Arabidopsis*, qRT-PCR, high-temperature stress, reference genes

## Abstract

Quantitative real-time PCR (qRT-PCR) is an essential tool for analyzing and selecting stable reference genes. In order to screen for suitable reference genes under high-temperature stress conditions in *Arabidopsis*, this study measured the relative expression levels of 17 candidate reference genes using qRT-PCR. Among these, four are traditional reference genes, while the remaining thirteen are candidate reference genes with no previous reports on their use as reference genes. The expression stability of the candidate reference gene expression was analyzed and evaluated using five methods: ΔCt, geNorm, NormFinder, BestKeeper, and RefFinder. The results indicated that the *LHCB4.1* and *LHCB5* genes displayed the highest stability in expression under high-temperature stress conditions. To verify the stability of the reference genes, we treated *Arabidopsis* with high-temperature stress, used the selected *LHCB4.1* and *LHCB5* as references, and analyzed the expression of the heat-responsive gene *HSFA2* using qRT-PCR. The results showed that when *LHCB4.1* and *LHCB5* were used individually or in combination as reference genes, the relative expression of *HSFA2* significantly increased and remained consistent under high-temperature treatment. This indicates that both *LHCB4.1* and *LHCB5* are suitable reference genes for qRT-PCR analysis in *Arabidopsis* exposed to high-temperature stress. The novel reference genes identified in this study will serve as a reliable reference standard for gene expression studies in *Arabidopsis* under high-temperature stress, thereby enhancing the accuracy and comparability of experimental data.

## 1. Introduction

As global warming intensifies, extreme heatwaves are increasingly frequent. High-temperature stress occurs when plants encounter temperatures higher than their optimal growth range, which may inhibit or damage plant growth and development [1]. This stress can result in various impacts, such as reduced photosynthesis, cell membrane damage, and metabolic disorders, with effects that can vary among different plant species and conditions [2,3]. Plants have developed a series of complex response mechanisms to cope with high-temperature stress during their long-term evolution, including the synthesis of heat shock proteins, the activation of antioxidant systems, and the accumulation of osmotic regulatory substances. These response mechanisms are mediated by complex regulatory networks that involve the coordinated control of multiple genes [4]. *Arabidopsis thaliana*, a model plant, is widely utilized for research in genetics, biochemistry, and molecular mechanisms. Over the years, significant strides have been made in the field of plant stress tolerance, particularly in molecular genetics research using *Arabidopsis thaliana* as a model organism for elucidating plant responses to various abiotic stresses, including drought, salinity, and heat stress [5]. Amid escalating global climate change, research on stress-responsive genes has increasingly become a focal point within the scientific community. Therefore, the precise detection and analysis of stress-responsive gene expression patterns are crucial for gaining an in-depth understanding of the molecular mechanisms underlying plant stress responses.

Quantitative real-time PCR (qRT-PCR) is a technique derived from traditional PCR and serves as a pivotal tool for assessing gene expression in plant molecular biology research. This technology enables the real-time quantification of DNA amplification by monitoring the fluorescence signal throughout the PCR cycle and utilizes a reference gene to standardize and precisely quantify the relative expression levels of specific target genes in a comparative manner among different samples. This methodology offers several key advantages, including high specificity, high sensitivity, excellent repeatability, and enhanced quantitative precision [6]. By selecting reference genes characterized by their consistent expression across various conditions or tissues, we use them as reliable internal controls in qRT-PCR, which allows the experimental data to be normalized. This process mitigates the impact of variables such as the initial RNA template concentration, reverse transcription efficiency, and PCR amplification efficiency, thereby ensuring the reliability and accuracy of the gene expression measurements [7]. Ideally, a reference gene should exhibit stable expression across various plant organs, developmental stages, and environmental conditions [8]. However, to date, no such universally applicable reference gene has been identified [9]. Research has indicated that during the flowering process of rice (*Oryza sativa* L.), the gene encoding eukaryotic elongation factor 1-alpha (eEF1-α) exhibits the most stable expression in japonica rice varieties. Conversely, the gene encoding ubiquitin-conjugating enzyme (UBC) demonstrates the highest stability in indica rice varieties. As a result, these genes are considered suitable reference genes for their respective rice subspecies [10]. For the physic nut (*Jatropha curcas*), distinct reference gene pairs are necessary at various developmental stages. The combination of *ACTIN* and *TUBULIN BETA 8* (*TUB8*) is optimal during the vegetative growth phase, whereas *GLYCERALDEHYDE-3-PHOSPHATE DEHYDROGENASE* (*GAPDH*) and *EF-1α* are more suitable for the reproductive growth phase [11]. In cotton (*Gossypium hirsutum* L.), *GAPDH* and *EF1α-8* exhibit stable expressions in both leaf and root tissues under salt stress, making them suitable reference genes for normalization. However, under drought stress, their expression levels are downregulated, which disqualifies them as reliable reference genes for such conditions [12]. These studies collectively suggest that the selection of reference genes should be tailored and optimized based on specific experimental conditions to ensure the precision and reliability of the experimental data.

In *Arabidopsis*, traditional reference genes such as *ACTIN*, *UBIQUITIN*(*UBQ*), and *GAPDH* are commonly employed [13,14,15]. To identify optimal reference genes under high-temperature stress conditions in *Arabidopsis*, we performed a comprehensive transcriptomic profiling analysis of *Arabidopsis* exposed to elevated temperatures. This analysis resulted in the identification of 13 novel candidate reference genes, which could potentially serve as robust internal controls for gene expression studies under thermal stress conditions. Using qRT-PCR technology, we initially applied the ΔCt methodology to ascertain the expression levels of 13 novel candidate reference genes and four traditional reference genes. Subsequently, we utilized three bioinformatics algorithms, geNorm [16], NormFinder [17], and BestKeeper [18], to assess the expression stability of these candidate reference genes under high-temperature stress conditions. Ultimately, an integrated evaluation was conducted using the RefFinder [19] platform. These five algorithms each have their own advantages: ΔCt: The experimental design is simple, data analysis and processing are straightforward, and it is easy to operate. geNorm: It can handle large-scale gene expression data; it determines the optimal number of reference genes and their stability. NormFinder: It takes into account both inter-sample and intra-sample variation factors, and its assessment results may be better than other software. BestKeeper: The operation is simple, and combining multiple candidate reference genes forms an index that can more comprehensively evaluate the performance of reference genes. RefFinder: It integrates the ΔCt, geNorm, NormFinder, and BestKeeper methods to assess the stability and reliability of reference genes. These five algorithms are currently widely used. Using these five algorithms, we have determined that the genes *LHCB4.1* and *LHCB5* are the most stably expressed under high-temperature stress, making them suitable as reference genes for *Arabidopsis* under high-temperature stress conditions. The aim of this study is to provide stable reference genes for the research of stress-resistant genes in *Arabidopsis* under high-temperature stress, laying the foundation for the accurate analysis of the expression levels of related genes in later studies.

## 2. Materials and Methods

### 2.1. Experimental Materials

*Arabidopsis thaliana* ecotype Col-0 plants were cultivated at 22 °C under a long-day (LD) photoperiod with a 16 h light/8 h dark cycle. The photosynthetic photon flux density was maintained at 160 μmol m^−2^ s^−1^, using T5 fluorescent lamps from Philips.

### 2.2. High-Temperature Stress Treatment

*Arabidopsis thaliana* ecotype Col-0 plants were cultivated under a 22 °C photoperiod in a growth chamber for three weeks. Subsequently, the plants were exposed to high-temperature stress treatments with the following conditions: 1. Control group maintained at 22 °C; 2. Acute heat treatment at 42 °C for 2.5 h; 3. Chronic heat treatment at 32 °C for 48 h. Following treatment, leaf tissues were harvested from each group, swiftly immersed in liquid nitrogen to flash freeze, and then stored at −80 °C for preservation. These samples were prepared for subsequent RNA extraction and gene expression analysis.

### 2.3. Total RNA Extraction and cDNA Synthesis

Isolate 100 mg of leaf tissue from each treatment group and extract total RNA from *Arabidopsis* using the Trizol reagent, which is procured from Nanjing Vazyme Biotech Co., Ltd. Assess RNA purity and concentration by determining the optical density (OD) at 260 nm and 280 nm with a nanodrop spectrophotometer (Thermo Scientific, Waltham, MA, USA). Ensure that the OD260/OD280 ratio of all samples falls within the range of 1.8 to 2.0, signifying high nucleic acid purity. Verify the integrity of the RNA through 1% agarose gel electrophoresis, where distinct bands without diffuse smears confirm the absence of degradation. Subsequently, perform reverse transcription of the purified RNA to synthesize complementary DNA (cDNA) utilizing the HiScript^®^ II 1st Strand cDNA Synthesis Kit (Nanjing Vazyme Biotech Co., Ltd., Nanjing, China). Store the synthesized cDNA at −20 °C for subsequent applications.

### 2.4. Identification of Novel Candidate Reference Genes

We retrieved RNA-seq data pertaining to *Arabidopsis* under heat stress conditions from the NCBI Gene Expression Omnibus (GEO) database, accession number GSE200247. We then computed the average FPKM, standard deviation (SD), and coefficient of variation (CV) for each gene [19].

To identify genes with high expression levels and stability, we selected genes with an average FPKM value exceeding 150 and ranked them based on their standard deviation (SD) values, from the lowest to the highest. Then, we further filtered the top 350 genes based on a CV value below 0.3. Subsequently, we identified 13 candidate genes based on their tissue expression distribution, which exhibited high abundance across various tissues. In addition to the 13 reference genes screened, this study also chose 4 traditional reference genes as controls, which are as follows: *GAPDH*, *UBQ5*, *ACTIN2*, and *UBQ10* (Table 1).

### 2.5. Primer Design and Quantitative Real-Time PCR

Utilize the GenScript Real-Time PCR TaqMan Primer Design Tool (https://www.genscript.com.cn/tools/real-time-pcr-taqman-primer-design-tool, accessed on 5 January 2024) to design primers with the following specifications: target amplicon length between 100 and 150 base pairs, primer length ranging from 18 to 22 nucleotides, GC content within the range of 45% to 55%, and melting temperature (Tm) between 55 and 60 °C. For detailed specifications, refer to Table 2.

The experiment employs a Bio-Rad CFX96 Touch Real-Time PCR Detection System and ChamQ^TM^ SYBR qPCR Master Mix from Nanjing Vazyme Biotech Co., Ltd. The reaction mixture has a total volume of 20 μL, comprising 10 μL of ChamQ^TM^ SYBR qPCR Master Mix, 0.2 μL each of forward and reverse primers at a concentration of 10 μM, 3 μL of template cDNA, and 6.6 μL of nuclease-free water. The thermal cycling conditions are as follows: initial denaturation at 95 °C for 3 min; followed by 40 cycles of denaturation at 95 °C for 10 s, annealing at 56 °C for 30 s, and extension at 72 °C for 30 s with fluorescence signal acquisition; and a final step for the collection of the melting curve fluorescence signal.

### 2.6. Stability Verification of Internal Reference Genes

To assess the stability of reference genes under the conditions of heat stress, we employed the comparative CT (2^−ΔΔCt^) method to quantify the relative expression levels of the genes under investigation [20]. Furthermore, we utilized three software programs known for their gene stability analysis capabilities: geNorm, NormFinder, and BestKeeper, in conjunction with the web-based tool RefFinder (https://blooge.cn/RefFinder/, accessed on 7 March 2024.), to conduct a comprehensive assessment of the stability of 17 candidate reference genes. This analysis aimed to identify the most stable reference gene for *Arabidopsis* under high-temperature stress conditions.

## 3. Results

### 3.1. RNA Quality Assessment

**Figure 1:**
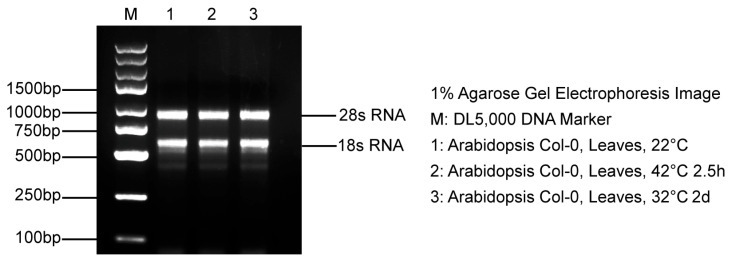
The results of agarose gel electrophoresis of total RNA.

Following purification, RNA extracted from triplicate samples was evaluated for integrity using agarose gel electrophoresis. The resulting electrophoretic profiles displayed distinct bands corresponding to the 28S and 18S ribosomal RNAs, confirming the integrity of the RNA and the absence of significant degradation (Figure 1). Further purity assessment conducted with a nanodrop spectrophotometer demonstrated OD260/OD280 ratios ranging from 1.8 to 2.0, and OD260/OD230 ratios exceeding 2.0, both of which are hallmarks of high-quality RNA. The absence of additional peaks in the spectrophotometric trace corroborated the absence of protein contamination. These findings ensured that the RNA samples were of sufficient purity and integrity for subsequent experimental applications.

### 3.2. Verification of the Specificity of Primers for Candidate Reference Genes

**Figure 2:**
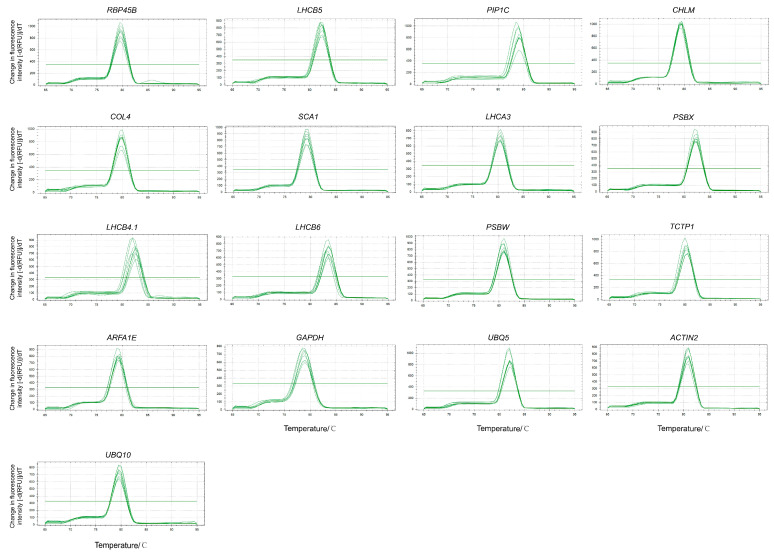
Melting curves of qPCR amplification products for 17 candidate reference genes.

Analysis of the melting curves from real-time fluorescent quantitative PCR (qPCR) indicated that each candidate reference gene exhibited a single specific melting peak, with no detectable side peaks (Figure 2). This finding suggests that the primers have high amplification specificity, ruling out the presence of primer dimers or non-specific amplification products. Consequently, these primers are deemed appropriate for use in subsequent qRT-PCR experiments. Based on these results, 17 candidate genes were selected for further analysis, including *RBP45B*, *LHCB5*, *PIP1C*, *CHLM*, *COL4*, *SCA1*, *LHCA3*, *PSBX*, *LHCB4.1*, *LHCB6*, *PSBW*, *TCTP1*, *ARFA1E*, *GAPDH*, *UBQ5*, *ACTIN2*, and *UBQ10*.

### 3.3. Analysis of the Stability of Candidate Reference Genes Under High-Temperature Stress

#### 3.3.1. Ct Value Analysis and ΔCt Value Calculation

In quantitative real-time PCR, the cycle threshold (Ct) value is defined as the number of cycles required for the fluorescence signal of the amplified product in each reaction tube to exceed a predetermined threshold. The Ct value serves as a quantitative measure of the initial template copy number of the target gene within the sample. A lower Ct value is indicative of a higher initial template concentration, thus reflecting a higher level of gene expression. Consequently, an inverse correlation exists between the Ct value and the relative expression level of the gene.

**Figure 3:**
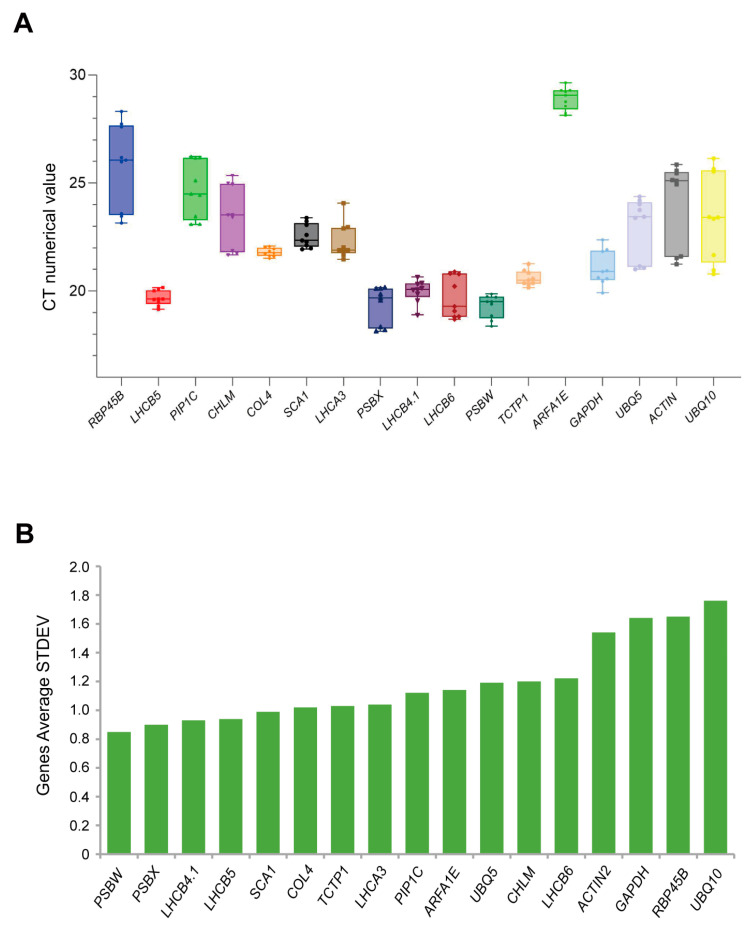
Analysis of cycle threshold (Ct) variability in candidate reference genes. (**A**) Boxplot of Ct values of 17 candidate genes. In terms of gene expression levels, *PSBW* has the highest expression level, while *ARFA1E* has the lowest. Regarding gene expression stability, *COL4* shows the most stable expression, whereas *UBQ10* and *RBP45B* exhibit relatively poor stability. (**B**) Expression stability was analyzed using the delta-CT method. *PSBW* has a low average standard deviation and a small coefficient of variation, indicating stable expression, while *UBQ10* has relatively poor stability.

We conducted qRT-PCR assays on samples to assess the expression levels and stability of 17 candidate reference genes under both normal growth conditions and high-temperature stress. The results revealed that the *PSBW* gene exhibited the lowest Ct value, indicating the highest level of expression. In contrast, the *ARFA1E* gene had the highest Ct value, suggesting the lowest level of expression. The Ct values for the other 15 genes varied between 18 and 28 cycles (Figure 3A). In terms of stability, the *COL4* gene showed the most stable expression, as indicated by the narrowest range in its boxplot (21.51–22.07). In contrast, the *UBQ10* and *RBP45B* genes exhibited wider boxplot ranges (20.79–26.14 and 23.15–28.31, respectively), suggesting less stable expression patterns (Figure 3A).

The ΔCt analysis ranks genes based on the average standard deviation (SD) of their Ct values, further revealing the expression stability of these candidate reference genes. The expression levels of *PSBW*, *PSBX*, *LHCB4.1*, *LHCB5*, *SCA1*, and *COL4* are relatively stable under high-temperature stress, as indicated by their low average standard deviation (SD) and small coefficient of variation. In contrast, the expressions of *ACTIN2*, *GAPDH*, *RBP45B*, and *UBQ10* are less stable, as evidenced by their high standard deviation and large coefficient of variation (Figure 3B). These results indicate that under the same conditions, there are variations in the expression levels among different candidate reference genes. Furthermore, under high-temperature stress, there are also significant differences in the expression stability of these genes themselves.

#### 3.3.2. geNorm Analysis

**Figure 4:**
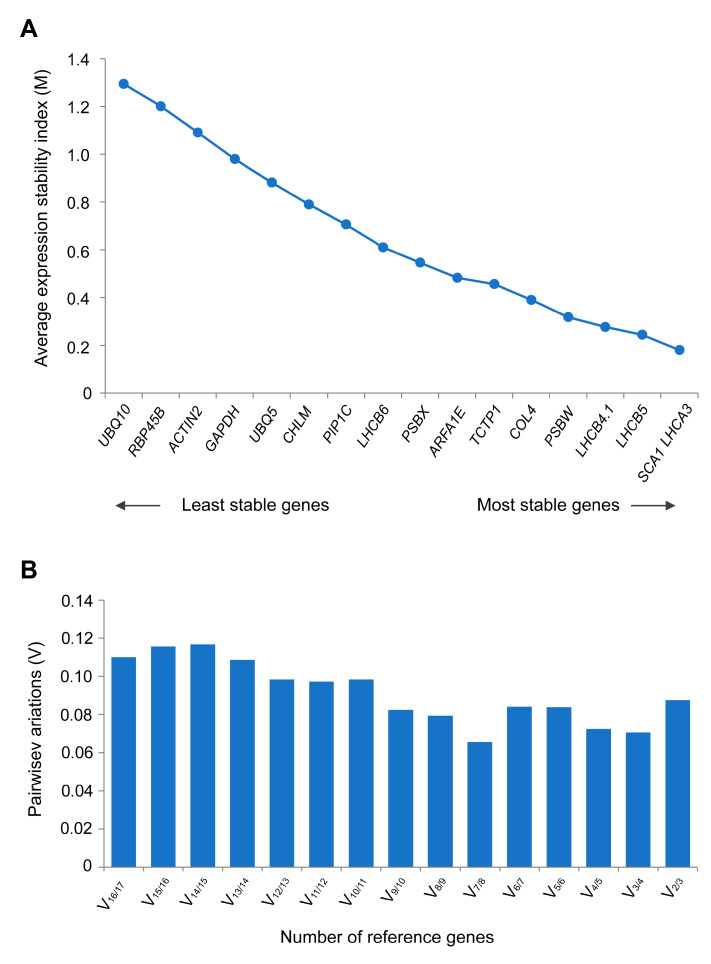
Stability analysis of candidate reference genes by geNorm. (**A**) geNorm assessment of expression stability depicted by M values, with stability increasing from left to right. *SCA1* and *LHCA3* have the smallest M values, indicating the best stability, while *UBQ10* has the poorest stability. (**B**) Pairwise variation analysis (Vn/n + 1) for optimal reference gene selection. Labeled as follows: 2: *LHCA3*; 3: *LHCB5*; 4: *LHCB4.1*; 5: *PSBW*; 6: *COL4*; 7: *TCTP1*; 8: *ARFA1E*; 9: *PSBX*; 10: *LHCB6*; 11: *PIP1C*; 12: *CHLM*; 13: *UBQ5*; 14: *GAPDH*; 15: *ACTIN2*; 16: *RBP45B*; 17: *UBQ10*.

The geNorm program evaluates the stability of reference genes using the M value, which represents the average expression stability index. A lower M value indicates more stable gene expression. If a gene’s M value is below 1.5, it can be considered a suitable candidate reference gene [16]. The analysis results indicate that under high-temperature stress treatment, all candidate reference genes have an M value below 1.5. Their stability is ranked from highest to lowest as follows: *SCA1* = *LHCA3 > LHCB5 > LHCB4.1 > PSBW > COL4 > TCTP1 > ARFA1E > PSBX > LHCB6 > PIP1C > CHLM > UBQ5 > GAPDH > ACTIN2 > RBP45B > UBQ10*. Based on the M value, the top five candidate reference genes are *SCA1*, *LHCA3*, *LHCB5*, *LHCB4.1*, and *PSBW*, indicating that these genes exhibit greater stability in expression than the others (Figure 4A, Table 3).

The geNorm algorithm’s V value represents the pairwise variation, which is used to determine the optimal number of reference genes. If the ratio of Vn/Vn + 1 is below 0.15, then using n reference genes is considered optimal; otherwise, n + 1 reference genes are required [21]. As depicted in Figure 4B, the V_2_/V_3_ value for this study is 0.087, which is less than 0.15, indicating that the combination of the two most stable reference genes suffices for the calibration requirements of the qRT-PCR data.

#### 3.3.3. NormFinder Analysis

**Figure 5:**
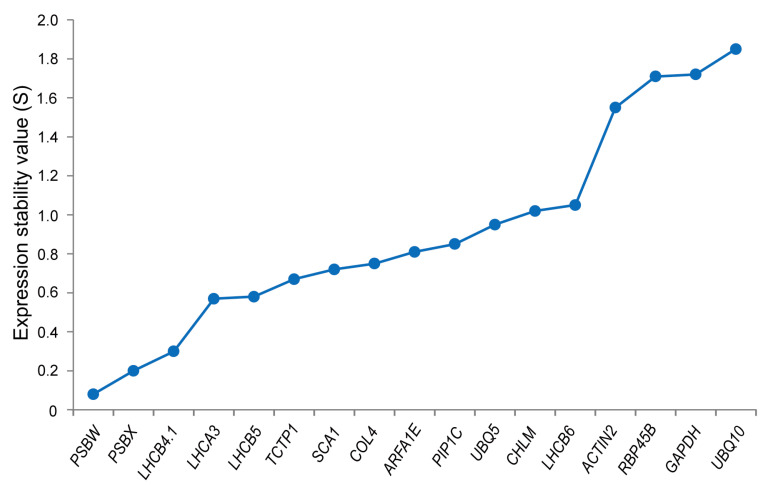
NormFinder analyzes the expression stability of internal reference genes under high-temperature stress. The stability of candidate internal reference genes decreases from left to right. *PSBW* has the smallest S value, indicating the best stability, while *UBQ10* has the worst stability.

The NormFinder software ranks gene stability based on the expression stability value (S value), where a smaller S value signifies more stable gene expression [17]. Under high-temperature stress treatment, Figure 5 and Table 3 display the S values of the 17 candidate reference genes, with their stability ranked from highest to lowest. Among them, *PSBW*, *PSBX*, *LHCB4.1*, *LHCA3*, and *LHCB5* exhibit the smallest S values, suggesting that these five candidate genes are more stably expressed. In contrast, *UBQ10* and *RBP45B*, with their higher S values, are less stable. In addition, observations indicate that the new reference genes generally exhibit higher stability rankings compared to the traditional reference genes.

#### 3.3.4. BestKeeper Analysis

**Figure 6:**
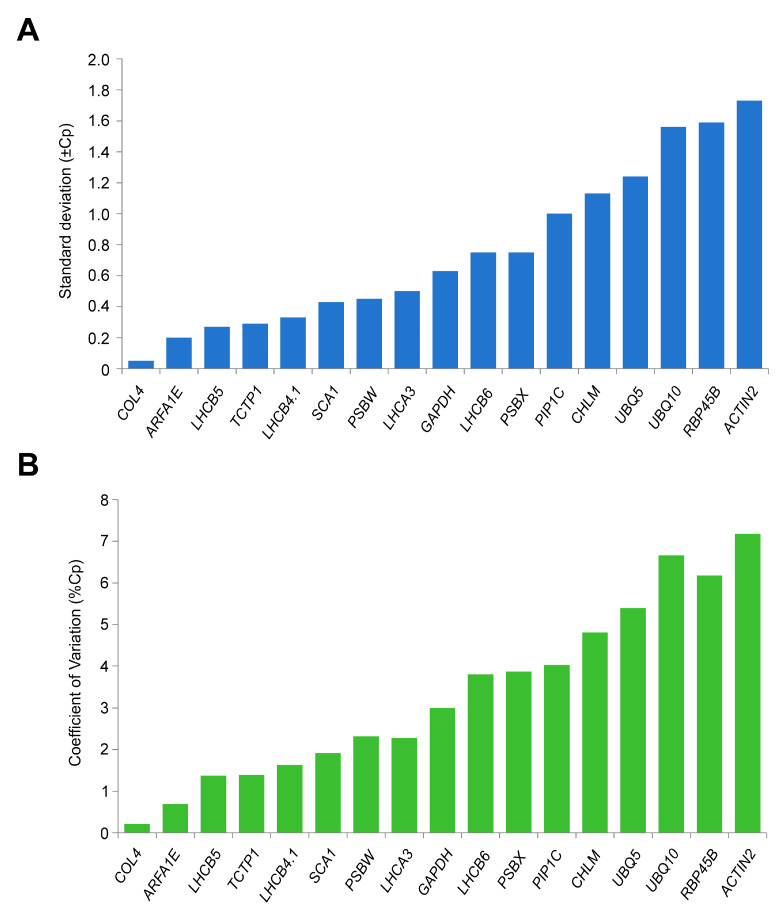
BestKeeper was used to evaluate the expression stability of 17 reference genes under high-temperature stress. (**A**) SD values of 17 candidate internal reference genes. *COL4* has the smallest SD value, indicating the best stability, while *ACTIN2* has the poorest stability. (**B**) CV values of the 17 candidate internal reference genes. *COL4* has the lowest coefficient of variation (CV), indicating the best stability, while *ACTIN2* has the poorest stability.

The BestKeeper software primarily evaluates gene stability by comparing the standard deviation (SD) and coefficient of variation (CV) of the Ct values among candidate reference genes. Lower SD and CV values indicate higher gene expression stability. When the SD value exceeds 1.0, the gene is not suitable for use as a reference gene [18]. Experimental results indicate that under high-temperature stress, the genes *COL4*, *ARFA1E*, *LHCB5*, *TCTP1*, and *LHCB4.1* exhibit greater stability. Conversely, *PIP1C*, *CHLM*, *UBQ5*, *UBQ10*, *RBP45B*, and *ACTIN2*, with SD values above 1.0, are deemed unsuitable as reference genes under these conditions, as illustrated in Figure 6A,B, Table 3.

#### 3.3.5. RefFinder Analysis

To minimize potential errors from relying on a single evaluation software, we utilized the RefFinder analytical tool. This online tool calculates the geometric mean of gene expression stability rankings from three software programs—geNorm, NormFinder, and BestKeeper—under high-temperature stress, offering a comprehensive ranking. The lower the geometric mean, the more stable the gene expression is deemed [19].

**Figure 7:**
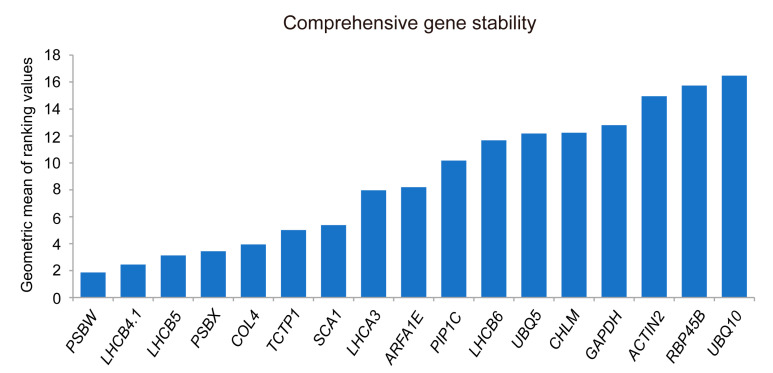
RefFinder integrates and evaluates the stability of 17 candidate reference genes under high-temperature stress. *PSBW* has the smallest geometric mean, indicating the best stability, while *UBQ10* has the worst stability.

The analysis results show that the comprehensive stability ranking of the candidate reference genes from highest to lowest are as follows: *PSBW* > *LHCB4.1* > *LHCB5* > *PSBX* > *COL4* > *TCTP1* > *SCA1* > *LHCA3* > *ARFA1E* > *PIP1C* > *LHCB6* > *UBQ5* > *CHLM* > *GAPDH* > *ACTIN2* > *RBP45B* > *UBQ10* (Figure 7 and Table 3).

### 3.4. Determination and Validation of Reference Genes

**Figure 8:**
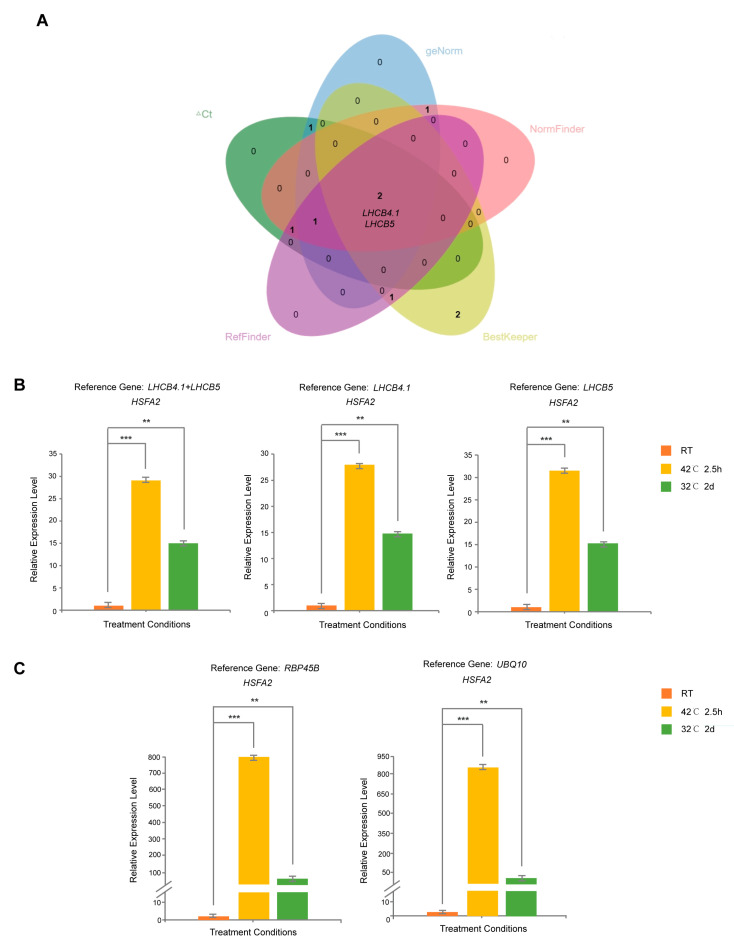
Validation of new internal reference genes. (**A**) Venn diagram representing the consensus of the top five genes across ΔCt, geNorm, NormFinder, BestKeeper, and RefFinder assessments. The two genes consistently identified by all methods are *LHCB4.1* and *LHCB5*. (**B**) *LHCB4.1* and *LHCB5* and their combinations were used as internal reference genes to correct the expression level of *HSFA2* under high-temperature treatment. (**C**) Observation of *HSFA2* expression level under high-temperature stress treatment using the unstable reference genes *RBP45B* and *UBQ10.* Note: ** indicates *t*-test result <0.01, and *** indicates significant difference in *t*-test result <0.001.

We ranked the candidate reference genes using five different methods and determined the most stable ones by finding the intersection (Figure 8A). The results indicate that *LHCB4.1* and *LHCB5* consistently exhibited high stability under heat stress across all five evaluation methods. To verify the accuracy of the software analysis results, we selected *Heat Stress Transcription Factor A2* (*HSFA2*) as the target gene for validation. *HSFA2* is a key heat shock transcription factor, exhibiting the highest expression level under high-temperature stress among the 21 members of the *Arabidopsis HSF* family [22]. It is highly sensitive to heat stress. We utilized *LHCB4.1* and *LHCB5* as reference genes, both individually and in combination, to evaluate *HSFA2* expression under high-temperature stress. Meanwhile, *RBP45B* and *UBQ10*, characterized as less stable by a thorough RefFinder analysis, functioned as negative control genes. The results showed that when *LHCB4.1* and *LHCB5*, individually or in combination, were used as reference genes, *HSFA2* expression was significantly upregulated under two high-temperature stress induction conditions (42 °C for 2.5 h; 32 °C for 2 d) compared to normal conditions. The upregulation under the 42 °C for 2.5 h treatment was approximately twice that observed under the 32 °C for 2 d treatment. Specifically, when *LHCB4.1* was used as a reference gene, its ΔCt values under the two high-temperature treatments were minimal, being 0.52 and 0.95, respectively. This resulted in a 28.0-fold and 14.8-fold upregulation in *HSFA2* expression under these conditions. Likewise, when *LHCB5* was used, its ΔCt values were even lower, at 0.08 and 0.65, respectively, corresponding to a 31.6-fold and 15.3-fold upregulation in *HSFA2* expression under the same conditions (Figure 8B). In contrast, when *RBP45B* and *UBQ10* were used as reference genes, the expression level of *HSFA2* was also significantly upregulated. However, the larger changes in their ΔCt values under the two high-temperature stress induction conditions—2.68 and 4.5 for *RBP45B*, and 2.25 and 4.64 for *UBQ10*—resulted in a much higher upregulation in *HSFA2*. Specifically, with *RBP45B* as the reference, *HSFA2* expression was upregulated by approximately 769.0-fold and 62.8-fold under the respective conditions. With *UBQ10* as the reference, the upregulation was about 853.2-fold and 46.6-fold. The difference in the upregulation fold-change in *HSFA2* expression between the two stress conditions was about 13-fold and 18-fold (Figure 8C).

Therefore, the large variation in the original Ct values of *RBP45B* and *UBQ10* before and after high-temperature stress induction led to high and less reliable expression level values for *HSFA2*. The large differences in these values diminished their credibility, making them unsuitable as reference genes. In summary, *LHCB4.1*, *LHCB5*, and their combined use demonstrated greater stability under high-temperature stress, rendering them ideal reference genes for such conditions. This insight will facilitate future research on gene function under high-temperature stress in *Arabidopsis*.

## 4. Discussion

High-temperature stress is one of the abiotic stresses that plants encounter during their growth and development. Temperatures of varying intensity and duration can inflict damage of differing severity at various stages of plant growth. *Arabidopsis thaliana*, a widely used model plant globally, offers an ideal subject for plant genetics research. Therefore, conducting in-depth studies on the gene expression regulation network in *Arabidopsis* under high-temperature stress, as well as exploring heat-resistant genes and molecular mechanisms, holds great scientific value for understanding plant stress response mechanisms. Furthermore, these studies not only establish an important scientific foundation but also have promising implications for developing crops that are resistant to high-temperature stress in agricultural production.

The accurate detection and analysis of heat-resistant gene expression patterns are essential to understanding how plants respond to and adapt to high-temperature stress. qRT-PCR technology has become an important tool in the scientific research of plants due to its extensive application in gene expression analysis. The stability of reference genes is crucial for the accuracy of qRT-PCR data analysis, allowing suitable reference genes to effectively correct experimental data and minimize errors. Nonetheless, research has demonstrated that no ideal reference genes consistently maintain stable expression. This study provides an in-depth investigation for the purpose of screening suitable reference genes in *Arabidopsis* under high-temperature stress. We initially screened for candidate reference genes with stable expression under high-temperature stress using transcriptome data analysis. Subsequently, we integrated these with traditional reference genes and utilized five algorithms (ΔCt, geNorm, NormFinder, BestKeeper, and RefFinder) to evaluate their stability under high-temperature stress. In conclusion, *LHCB4.1* and *LHCB5* demonstrated the most stable expression with the highest comprehensive ranking. Conversely, *LHCB6*, *UBQ5*, *CHLM*, *GAPDH*, *ACTIN2*, *RBP45B,* and *UBQ10* had a lower comprehensive ranking, with their stability decreasing in that order.

*LHCB4.1* and *LHCB5* are both members of the LHCB family, which encodes the light-harvesting chlorophyll a/b-binding proteins of Photosystem II (PSII). *LHCB4.1* and *LHCB5* are small chlorophyll-binding antenna proteins that play a crucial role in the photoprotection of Photosystem II (PSII). These proteins can safely dissipate excess excitation energy when light energy exceeds the photosynthetic system’s processing capacity, thereby safeguarding the plant from damage by reactive oxygen species generated during photosynthesis [23].

When analyzing the expression distribution of *LHCB4.1* and *LHCB5* across the various tissues of *Arabidopsis*, it was observed that these two genes exhibit their highest expression in rosette leaves, with significant levels also detected in seedlings, hypocotyls, cauline leaves, flowers, and siliques (Figure S1A,B). This pattern indicates that *LHCB4.1* and *LHCB5* can serve as reliable internal reference genes under high-temperature stress conditions in these tissues. However, their minimal expression in roots and mature pollen renders them less suitable as internal reference genes for these specific tissues (Figure S1A,B).

*UBQ10* encodes the polyubiquitin protein, which is primarily involved in the ubiquitination and degradation of proteins [24]. Tissue expression distribution indicates that *UBQ10* is consistently and highly expressed across all tissues and developmental stages, with the exception of mature pollen and late embryonic development, where its expression levels peak (Figure S1C). The ubiquitous expression of *UBQ10* renders it a popular internal reference gene for gene expression analysis, facilitating the standardization of experimental data and ensuring the accuracy of results. However, this study revealed that the expression stability of *UBQ10* diminishes under high-temperature stress, making it unsuitable as an internal reference gene for experiments conducted under these conditions.

Among the new candidate reference genes identified in this study, *RBP45B*, similar to *UBQ10*, has a low ranking and is therefore not recommended for use as a reference gene under high-temperature stress conditions. RBP45B is an RNA-binding protein that contains an RNA recognition motif (RRM). In *Arabidopsis*, it interacts with cap-binding protein 20 (CBP20) and polyA-binding protein 8 (PAB8), playing a crucial role in mRNA processing, stability, and translation initiation [25].

Tissue expression distribution indicates that *RBP45B* is stably expressed across all tissues and developmental stages, except for the late stages of embryonic development (Figure S1D). In this context, the expression stability of *RBP45B* even exceeds that of the traditional reference gene *UBQ10*. However, despite its relatively stable expression, *RBP45B* does not exhibit an expression peak as high as that of *UBQ10*. Thus, while *RBP45B* is not suitable as a reference gene under high-temperature stress, it can serve as a reliable reference gene for the different tissues and growth stages of *Arabidopsis* under normal growth conditions.

## 5. Conclusions

In this study, we analyzed the transcriptome data of *Arabidopsis* under high-temperature stress, screening for 13 new candidate reference genes. We evaluated these candidates alongside four traditional reference genes using qRT-PCR technology to determine which genes are most suitable as reference genes after high-temperature stress. The results indicate that *LHCB4.1* and *LHCB5* are the most stable reference genes following high-temperature stress, providing an accurate tool and reliable reference standard for gene expression analysis under high-temperature conditions. These findings not only lay the groundwork for the further exploration of the functions of heat-resistant genes in *Arabidopsis* but also offer valuable insights for selecting heat-stable reference genes in other plant species.

## Figures and Tables

**Table 1:** Functional description of 17 candidate internal reference genes.

Gene Name	Functional Description
*RBP45B*	RNA-binding protein. Involved in mRNA processing, stability, and translation initiation.
*LHCB5*	Light-harvesting chlorophyll a/b binding proteins. Participates in the capture and transfer of light energy.
*PIP1C*	Major intrinsic proteins. Involved in plant growth and development, as well as the transport and regulation of water within the plant.
*CHLM*	Magnesium protoporphyrin IX methyltransferase. Involved in the biosynthesis process of chlorophyll.
*COL4*	CONSTANS-LIKE family. Involved in the photoperiodic flowering pathway and abiotic stress response.
*SCA1*	Ribosomal protein S5 family. A component of the ribosome, involved in the process of protein synthesis.
*LHCA3*	Light-harvesting chlorophyll a/b binding proteins. Participates in the capture and transfer of light energy, and is one of the important functional proteins in PSI.
*PSBX*	Photosystem II protein family. Maintains efficient electron transfer in PSII.
*LHCB4.1*	Light-harvesting chlorophyll a/b binding proteins. Participates in the capture and transfer of light energy.
*LHCB6*	Light-harvesting chlorophyll a/b binding proteins. Participates in the capture and transfer of light energy.
*PSBW*	Photosystem II protein family. Involved in stress response and the regulation of plant chlorophyll fluorescence.
*TCTP1*	Translationally controlled tumor protein 1. A significant growth regulator in *Arabidopsis*.
*ARFA1E*	ADP-ribosylation factor. Involved in the protein transport process from the endoplasmic reticulum to the Golgi apparatus.
*GAPDH*	Glyceraldehyde-3-phosphate dehydrogenase, belongs to a family of multifunctional proteins. It is an enzyme that plays a key role in the glycolysis process.
*UBQ5*	Ubiquitin protein. Involved in the ubiquitination process of proteins.
*ACTIN2*	Actin family protein. Participates in the formation of the cytoskeleton, cell division, cell growth, cell movement, and the transport of materials within the cell.
*UBQ10*	Ubiquitin protein. Involved in the ubiquitination and degradation of proteins.

**Table 2:** Design of primers for 17 candidate internal reference genes.

Gene Name	Gene ID	Primer Sequence (5′→3′)
*RBP45B*	AT1G11650	F: CGAGATCCGGACTCTTTGGA R: GACCGGTTTGCTTGTTACGA
*LHCB5*	AT4G10340	F: CTGAGGTTGTTCTCCTCGGT R: AGAAGAGCTCCTTGCTCAGG
*PIP1C*	AT1G01620	F: ATTGGAATCGTCGCCAAGTG R: ATCGGAACCTTCGTCCTTGT
*CHLM*	AT4G25080	F: CTGCTGCTATGGTTGCTGAA R: CACGTCGAGACATACAACGG
*COL4*	AT5G24930	F: CCGACGGTGAACGAGAATTG R: CCACTCCCACTTCCATCGAC
*SCA1*	AT2G33800	F: AGAAGTTGTTGCTGCTGTTCA R: TGGTGAAGCAGGTCTAAGCA
*LHCA3*	AT1G61520	F: TTGTGGTTTGCTTCATCGCA R: TCCTCCAGTACCTTCTGGGT
*PSBX*	AT2G06520	F: ACTTAACCAGACCCGTTCGT R: GGAGATACCGGTCAAAGCCT
*LHCB4.1*	AT5G01530	F: GAATGGCTTACCGGCGTTAC R: TGGAACTCGATGTAGCCGAT
*LHCB6*	AT1G15820	F: ATGGATCAGCTCAGCCTCTC R: CGTTAAAGGTGGTGGCAACT
*PSBW*	AT2G30570	F: CTCCTTCTCAAGCCTACCGT R: TGACTGAGACGTTTCCTTGC
*TCTP1*	AT3G16640	F: GGGTTACTGTGGGAGCTGTA R: TGGTTGCTCCTGAAGTCTGA
*ARFA1E*	AT3G62290	F: TCGATGCAGCTGGTAAGACT R: GACATCCCACACGGTAAAGC
*GAPDH*	AT1G13440	F: TTGGTGACAACAGGTCAAGCA R: AAACTTGTCGCTCAATGCAATC
*UBQ5*	AT3G62250	F: TCAAGCTTCAACTCCTTCTTT R: GTGGTGCTAAGAAGAGGAAGA
*ACTIN2*	AT3G18780	F: GTTCCAGCCCTCGTTTGTG R: CAAGTGCTGTGATTTCTTTGCTC
*UBQ10*	AT4G05320	F: AGGATGGCAGAACTCTTGCT R: TCCCAGTCAACGTCTTAACG

**Table 3:** Using the software geNorm, NormFinder, and BestKeeper (2004), the stability of 17 candidate reference genes was assessed and ranked.

Gene	geNorm		NormFinder		BestKeeper			RefFinder	
	M	Rank	SD	Rank	std [±CP]	CV [% CP]	Rank	Geometric Mean of Ranking Values	Rank
*PSBW*	0.317864897	4	0.08	1	0.448148148	2.324151973	7	1.86	1
*LHCB4.1*	0.276747099	3	0.3	3	0.325925926	1.62999185	5	2.45	2
*LHCB5*	0.243791248	2	0.58	5	0.26962963	1.370610939	3	3.13	3
*PSBX*	0.546236016	8	0.2	2	0.74962963	3.874499895	11	3.44	4
*COL4*	0.390385703	5	0.75	8	0.045925926	0.210723086	1	3.94	5
*TCTP1*	0.456361943	6	0.67	6	0.285185185	1.385365503	4	5.01	6
*SCA1*	0.180010288	1	0.72	7	0.431851852	1.912635533	6	5.38	7
*LHCA3*	0.180010288	1	0.57	4	0.504444444	2.275005011	8	7.97	8
*ARFA1E*	0.482993823	7	0.81	9	0.2	0.691722389	2	8.19	9
*PIP1C*	0.705859587	10	0.85	10	0.995555556	4.031677466	12	10.17	10
*LHCB6*	0.610471106	9	1.05	13	0.748148148	3.796207551	10	11.68	11
*UBQ5*	0.881314931	12	0.95	11	1.237037037	5.3943182	14	12.18	12
*CHLM*	0.789568282	11	1.02	12	1.126666667	4.805231732	13	12.24	13
*GAPDH*	0.979666168	13	1.72	16	0.632592593	2.999806804	9	12.8	14
*ACTIN2*	1.091030519	14	1.55	14	1.726666667	7.180482395	17	14.95	15
*RBP45B*	1.200799573	15	1.71	15	1.592592593	6.176297382	16	15.74	16
*UBQ10*	1.29414846	16	1.85	17	1.560740741	6.660660376	15	16.48	17

## Data Availability

This study retrieved RNA-seq data related to Arabidopsis under heat stress conditions from the NCBI Gene Expression Omnibus (GEO) database, with the accession number GSE200247. The access URL is: https://www.ncbi.nlm.nih.gov/geo/query/acc.cgi?acc=GSE200247, accessed on 21 November 2024.

## Supplementary Materials

The following supporting information can be downloaded at: https://www.mdpi.com/article/10.3390/cimb46120857/s1. Figure S1. The tissue distribution map of 17 candidate genes.
