# Supplementary figures and images for: Identification and Validation of qRT-PCR Reference Genes for Analyzing Arabidopsis Responses to High-Temperature Stress
Source: Curr Issues Mol Biol. 2024 Dec 18;46(12):14304–20. doi: 10.3390/cimb46120857 (PMC11674290; doi:10.3390/cimb46120857)

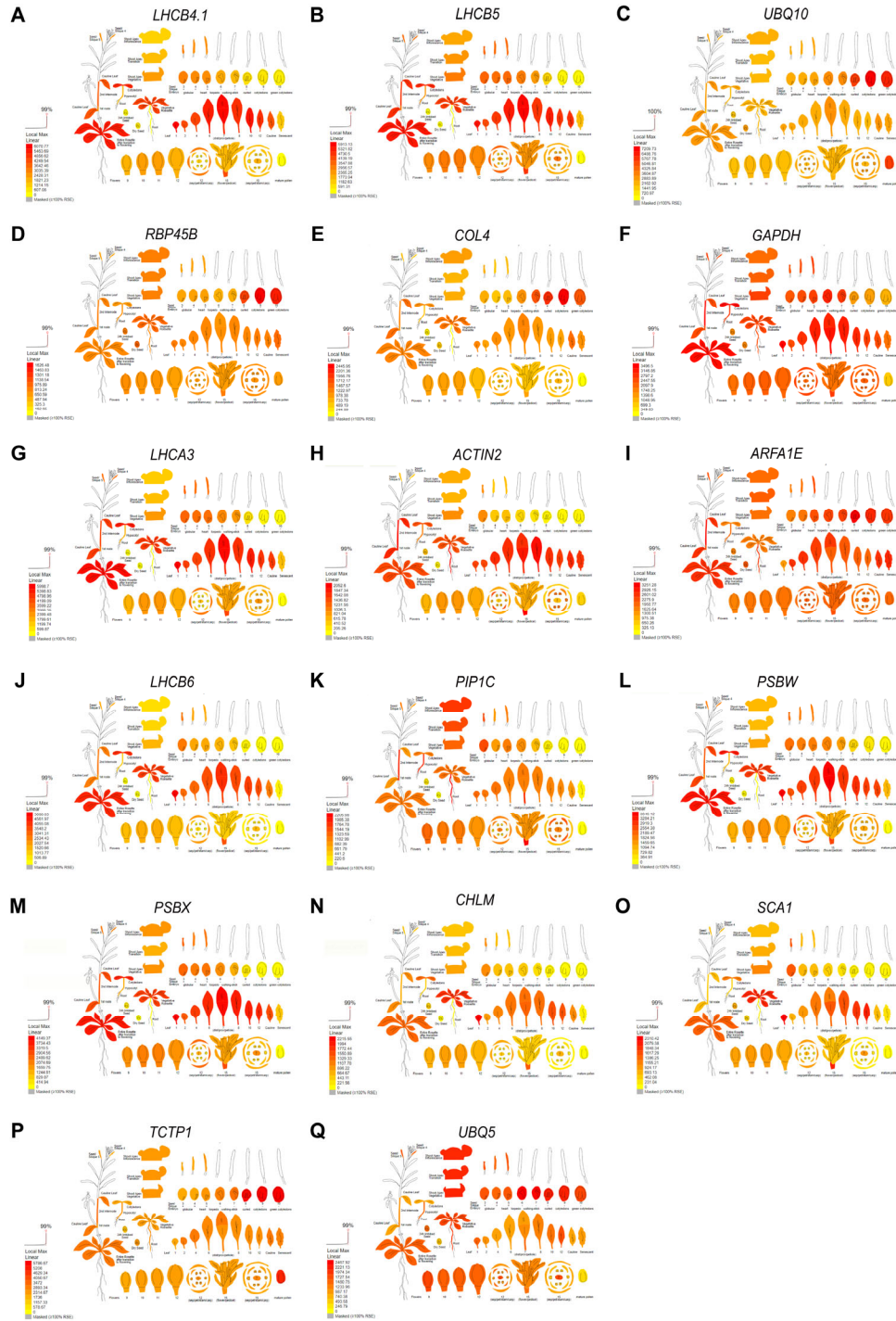

**Figure S1.** The tissue distribution map of 17 candidate genes.

Supplement: Supplementary file 1 [file cimb-46-00857-s001.zip › cimb-3357953-supplementary.pdf]
